# Evaluation of success rate of Zirconia based restorations: A systematic review

**DOI:** 10.4317/jced.59711

**Published:** 2022-09-01

**Authors:** Aditee Apte, Seema Sathe, Rewa Kawade

**Affiliations:** 1 Post Graduate Student, Department of Prosthodontics & Crown and Bridge, SPDC Sawangi (Meghe), Wardha; 2 Professor and Head of Department, Department of Prosthodontics & Crown and Bridge, SPDC Sawangi (Meghe), Wardha; 3 Post Graduate Student, Department of Prosthodontics & Crown and Bridge, SPDC Sawangi (Meghe), Wardha

## Abstract

**Background:**

Evaluation of the different causes listed in literature for the rate of success of Zirconia based restorations.

**Material and Methods:**

With the help of PRISMA guidelines , this Systematic review was carried out. For a time span of 18 years that is from 2003 to 2020, articles were searched using three electronic data bases which are PubMed , Cochrane Library and Sciencedirect. The selected 27 articles which included the in vivo as well as the in vitro studies presented the performance of zirconia-based prosthetic restorations. The studies also stated the commonest reason for failure which ultimately depicted the rate of success of the fixed dental prosthesis. Due to heterogeneity of gathered information , meta analysis could not be carried out.

**Results:**

Failure of bond between veneer material and zirconia sub-structure could be related to the cause of fracture of veneering porcelain hypothetically.

**Conclusions:**

Mechanical connection and building up of compressive strength due to thermal contraction at the time of cooling after sintering process is the reason for the bond developed amongst the two materials.

** Key words:**Zirconia based restorations, zirconia failure cause.

## Introduction

Esthetics satisfying the contemporary consideration for attractiveness is treated with the help of prosthodontic treatment by traditionally restoring the lost function of speech, chewing and deglutition. Social burden and welfare of the profession maximizes the conditioned necessity of esthetics (1).

The materials of choice for the cases in which esthetics is the key expectation are ‘Ceramics’ in recent times of which ‘yttria-stabilized tetragonal zirconia polycrystals’ (Y-TZP) is the most advanced core ceramic (1). This particular material enhanced high toughness and strength in multiple-unit FPDs.

Even if zirconia-based ceramics being a prime material for fabricating FPDs, its high resistance to fracture could also endure high occlusal loads adding a major advantage.

Nonetheless, cohesive fractures of the veneering ceramic is a ‘weak link’ of the restoration seen as the short-term clinical letdowns of zirconia-based restorations (1).

## Material and Methods

-Review Question

Population – *In vivo* as well as *in vitro* studies performed with zirconia based restorations

Intervention – Studies with success rate of anterior and posterior zirconia based restorations as FPDs or single crowns.

Outcome –Overall success rate of the zirconia based restorations

-Literature search

From 2003 to 2020, articles were searched using three electronic data bases which are PubMed , Cochrane Library and Sciencedirect. Articles with full texts that contended the criteria for inclusion were attained. To include all relevant articles and for improving the electronic search, a final manual search was carried out amongst the selected articles to get cross references and citations.

PubMed provided 114 articles and Science direct provided 8 articles and citation search provided 17 articles after the electronic and manual search was done. So far , no systematic review has been published on the current topic. Total 80 articles were excluded and 59 articles were screened. These 59 articles were completely analyzed by the title and abstract leading to selection of only 27 relevant articles which served the criteria for inclusion considered for the systematic review.

## Results

-Results of data extraction

By gathering all the data after excluding the duplicates, full text of these 27 articles was attained lead by thorough screening of the remaining 59 articles. Therefore, for this systematic review 27 articles was the final sample size.

-Results of included studies

No inference has yet touched regarding the attempt to substitute the metal in metal ceramic restorations having ceramics of greater resistance. Its discussion began at the end of the 20th century. In current situations, Zirconium oxide the foremost target of research and trials held clinically. Chemical along with dimensional stability, mechanical resistance, hardness, and modulus of elasticity of the similar demand that of stainless steel are the primary characteristics supporting its usage as a biomaterial.

Chipping of veneers often goes overlooked by the patient and is simply corrected by intraoral polishing or repair inferring that it is an esthetic defect of slight status. This is the reason which leads the rate of survival of zirconia-based fixed dental prostheses and metal ceramic restorations equivalent upto 97 to 99% over a period of 5 years.

The greatest numbers of problems due to the usage of zirconium oxide in prosthetic conducts occur with fixed partial prostheses or fixed bridges. Various studies clinically showed cohesive type of fracture of the veneer material as a major and utmost liability. However, there is a debate as to the rate of occurrence of this mechanical letdown because of variations in the variables evaluated in various studies and the success rate of the prostheses have been calculated. They have been summarized in Table 1- Table 1 cont.-1.

**Table 1:**
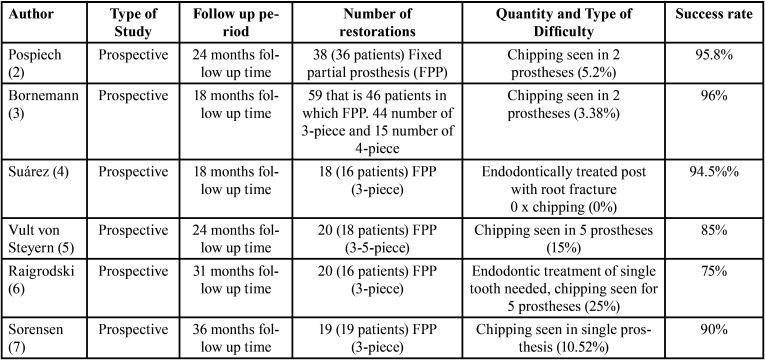
Success rate of prostheses calculated.

**Table 1 cont.**
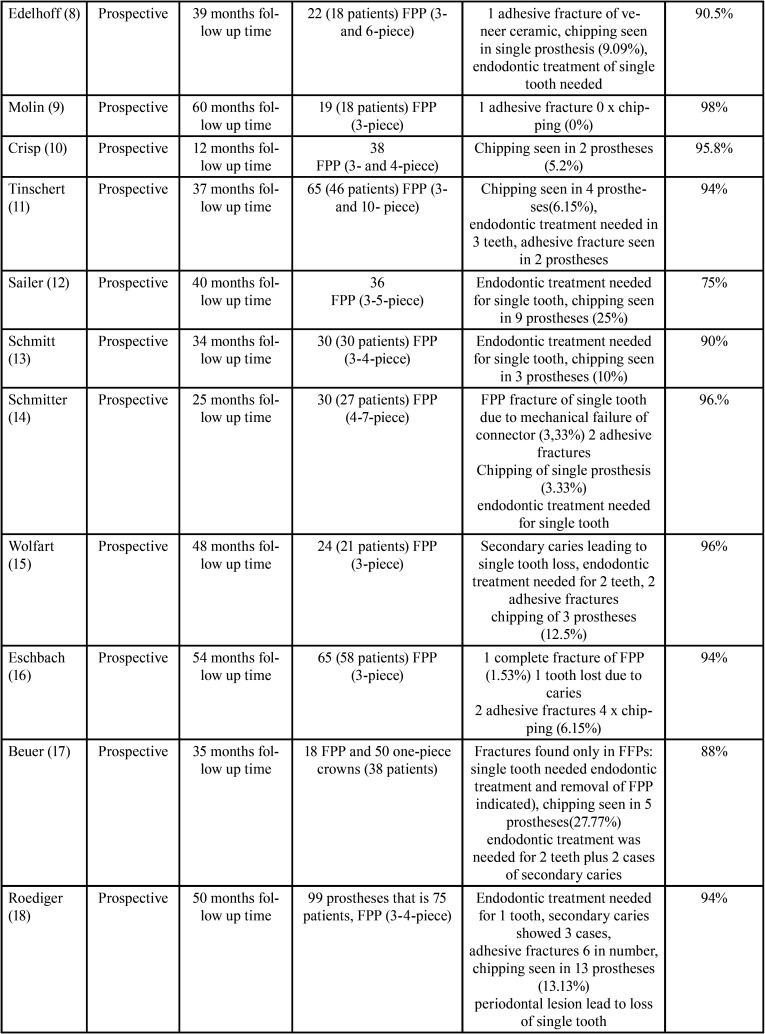
Success rate of prostheses calculated.

**Table 1 cont-1:**
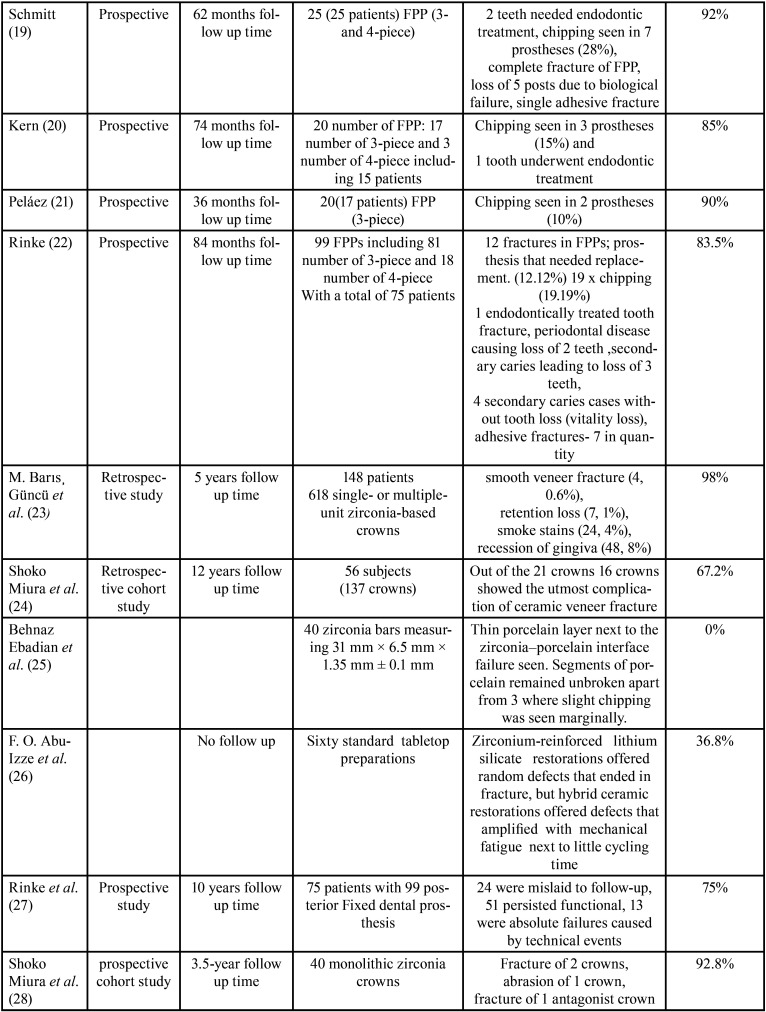
Success rate of prostheses calculated.

## Discussion

The studies were divided into groups - *In vivo* and *In vitro* studies.

-Fixed prostheses with zirconia substructure – *In vivo* performance in studies

The authors Pospiech (follow-up of 24 months) (2), Beuer (follow up of 40 months) (17), Bornemann (18 months) (3), Crisp (follow up of 12 months) (10), Tinschert (follow up of 37 months) (11), Schmitter (25 months follow up) (14), and Eschbach (follow up of 54 months) (16) evaluated chipping as the main cause of failure of the fixed prosthesis in their respective studies.

Vult von Steyern in his study with 24 months of follow up period (5), Peláez with follow up period of 36 months (21), Edelhoff (39 months of follow up)(8), Schmitt (follow up period of 34 months) (13), Wolfart (48±7 months of follow up time) (15), Roediger (50 months follow up)(18), Kern (follow up period of 74 months) (20), and Sorensen (follow up period of 36 months) (7) inferred an occurence of chipping that ranges between 9-15% with the success rate of 91% to 85% in posterior fixed partial prostheses.

Finally, diversity in the studies carried out by Raigrodski (with 31months follow-up) (6), Sailer (40.3±2.8 months of follow up period) (12), Beuer (with 35±14 months follow up time ) (17), Schmitt (62 months follow up) (19) and Rinke (follow up period of 84 months) (27) say that frequency of chipping of veneer material on posterior fixed partial prostheses is in the range of 19-28% with rate of success ranging from 72-81%.Few authors – Molin (with 60-month follow-up)(9) and Suárez (follow up period of 18 months)(4) did’nt spot any mechanical problems amongst the restorations considered (Table 1- Table 1 cont.-1)

-Fixed prostheses with zirconia substructure – *In vitro* performance studies

Concerning the mechanical performance of fixed prosthetic restorations, resisting the force of chewing deprived of getting fractured is the vital need. The first molar is exposed to a 300-800N force, although the anterior region is exposed to 60-200N of mastication force. Forces can exceed upto 1000N in few parafunctional cases (1).

Oblique fractures were seen in maximum of the studies where the direction of force is towards occlusion from gingiva, from the center of the connector to the pontic’s center (1).

Due to this reason a pontic fabrication area of 6-9mm2 is recommended by most of the authors.

Classification given by Konstantinos along with Agustin for the fracture types (29) :

Cohesive (chipping): Fracture without disturbing the interface of ceramic core.

Adhesive: Fracture occuring at the ceramic to core bond.

It is seen in various *in vitro* studies that when there is fracture in the samples, a cohesive fracture pattern is suffered in the occlusal zone which is adjacent to antagonist’s contact point.

Tsalouchou evaluated to static resistance loading of zirconia crowns which were 50 in quantity, analysing of the transversal plane by SEM analysis is done and also display that the most recurring type of fracture resulted as cohesive fracture(30).Similarly , Saito made a study of fracture resistance of porcelain-veneered of 72 samples with zirconia cores, concluding that the most recurring fracture type was cohesive fracture which comprises of 88.8% (31).

-Summary

The rate of success was significantly predisposed by site, with crowns seated in the molar area displaying further biological and technical difficulties than anterior crowns. Fractures of ceramic material were also knowingly influenced by site, with molar crowns showing knowingly more risk for these fractures than anterior crowns.

In vitro full-coverage restorations studies have seen a greater occurence of cohesive type of fracture for zirconia restorations. The higher incidence of chipping is explained in a study by Martin Rosentritt (2009) that assayed zirconia restoration fracture resistance, finding that all samples suffered cohesive fractures due to inadequate performance of the veneer material (32).

## Conclusions

The relationship between chipping phenomenon and risk factors occurring clinically, chiefly occlusal aspects, ought to be taken into consideration in upcoming prospective studies. Speciﬁc attention should be given by dental practitioners to clinical constraints when performing zirconia based restorations till an answer is found to enhance the mechanical resistance of the materials.

## References

[1] Agustín-Panadero R, Román-Rodríguez JL, Ferreiroa A, Solá-Ruíz MF, Fons-Font A (2014). Zirconia in fixed prosthesis. A literature review. J Clin Exp Dent.

[2] Rountree P, Pospiech (2018). Clinical evaluation of zirconia-based all ceramic posterior bridges: two-year results. J Dent Res.

[3] Bornemann G (2006). Prospective Clinical Trial with Conventionally Luted Zirconia-based Fixed Partial Dentures-18-month Results. J Dent Res.

[4] Suárez MJ, Lozano JFL, Salido MP, Martinez F (2004). Three-year clinical evaluation of In-Ceram Zirconia posterior FPDs. Int J Prosthodont.

[5] Vult von Steyern P, Carlson P, Nilner K (2005). All-ceramic fixed partial dentures designed according to the DC-Zirkon technique. A 2-year clinical study. J Oral Rehabil.

[6] Raigrodski AJ, Chiche GJ, Potiket N, Hochstedler JL, Mohamed SE, Billiot S (2006). The efficacy of posterior three-unit zirconium oxide based ceramic fixed partial dental prostheses: A prospective clinical pilot study. J Prosthet Dent.

[7] Sorensen J, Rusch R, Yokohama K (2010). Clinical study of CAD/ CAM generated Y-TZP posterior fixed partial dentures. J Dent Res.

[8] Edelhoff D D, Floriam B B, Weber V, Johnen C (2008). HIP zirconia fixed partial dentures-clinical results after 3 yearsof clinical service. Quintessence Int.

[9] Molin M, Karlsson S (2008). Five-year clinical prospective evaluation of zirconia-based Denzir 3-unit FPDs. Int J Prosthodont.

[10] Crisp R, Cowan A, Lamb J, Thompson O, Tulloch N, Burke F (2008). A clinical evaluation of all-ceramic bridges placed in UK general dental practices: first-year results. Br Dent J.

[11] Tinschert J, Schulze KA, Natt G, Latzke P, Heussen N, Spiekermann H (2008). Clinical behavior of zirconia- based fixed partial dentures made of DC Zirkon: 3-years results. Int J Prosthodont.

[12] Sailer I, Gottner J, Hammerle C (2009). Randomized controlled clinical trial of zirconia- ceramic posterior fixed dental prostheses: A 3-years Follow-up. Int J Prosthodont.

[13] Schmitt J, Holst S, Wichmann M, Reich S (2009). Zirconia Posterior Fixed Parcial Dentures: A Prospective Clinical 3-year Follow-up. Int J Prosthodont.

[14] Schmitter M, Mussotter K, Rammelsberg P, Stober T, Ohlmann B, Gabbert O (2009). Clinical performance of extended zirconia frameworks for fixed dental prostheses: two-year results. J Oral Rehabil.

[15] Wolfart S, Harder S, Eschbach S, Lehmann F, Kern M (2009). Four-year clinical results of fixed dental zirconia prostheses with zirconia substructures (Cercon): end abutments vs cantilever design. Eur J Oral Sci.

[16] Eschbach S, Wolfart S, Bohlsen F, Kern M (2009). Clinical Evaluation of All-Ceramic Posterior three-unit FDPs Made of In-Ceram Zirconia. Int J Prosthodont.

[17] Beuer F, Stimmelmayr M, Wolfgang G, Edelhoff D, Güth J F, Naumann M (2010). Prospective study of zirconia-based restorations: 3 year clinical results. Quintessence Int.

[18] Roediger M, Gersdorff N, Huels A (2010). Prospective evaluation of zir- conia posterior fixed partial dentures: four-year clinical results. Int J Prosthodont.

[19] Schmitt J, Goellner M, Lohbauer U, Wichmann M, Reich S (2012). Zirconia posterior fixed partial dentures: 5-year clinical results of a prospective clinical trial. Int J Prosthodont.

[20] Kern T, Tinschert J, Schley JS, Wolfart S (2012). Five-year clinical evaluation of all-ceramic posterior FDPs made of In-Ceram Zirconia. Int J Prosthodont.

[21] Peláez J, Cogolludo PG, Serrano B, Lozano JoseFL, Suárez MJ (2012). A prospective evaluation of zirconia posterior fixed dental prostheses: three- year clinical results. J Prosthet Dent.

[22] Rinke S, Gersdorff N, Lange K, Roediger M (2013). Prospective evaluation of zirconia posterior fixed partial dentures: 7- year clinical results. Int J Prosthodont.

[23] Güncü MB, Cakan Mehmet U, Senay Canay M (2015). Zirconia-Based Crowns Up to 5 Years in Function: A Retrospective Clinical Study and Evaluation of Prosthetic Restorations and Failures. Int J Prosthodont.

[24] Miura S, Kasahara S, Yamauchi S, Okuyama Y, Izumida A, Aida J (2018). Clinical evaluation of zirconia-based all-ceramic single crowns: an up to 12-year retrospective cohort study. Clin Oral Invest.

[25] Ebadian B, Mosharraf R, Abbasi M (2018). Effect of ceramic cooling protocols and zirconia coloring on fracture load of zirconia‑based restorations. Dent Res J.

[26] Abu-Izze FO, Ramos GF, Borges ALS, Anami LC, Bottino MA (2018). Fatigue behavior of ultraﬁne tabletop ceramic restorations. J Dent Mat.

[27] Rinke S, Wehle J, Schulz X, Bürgers R, Rödiger M (2018). Prospective Evaluation of Posterior Fixed Zirconia Dental Prostheses: 10-Year Clinical Results. Int J Prosthodont.

[28] Miura S, Yamauchi S, Kasahara S, Katsuda Y, Fujisawa M, Egusa H (2021). Clinical evaluation of monolithic zirconia crowns: a failure analysis of clinically obtained cases from a 3.5-year study. J Prosthodont Res.

[29] Konstantinos XM, Athanasios S, Hirayama H, Kiho K, Foteini T, Yukio O (2009). Fracture resistance of metal ceramic restorations with two different margin designs after exposure to masticatory simulation. J Prosthet Dent.

[30] Tsalouchou E, Cattell MJ, Knowles JC, Pittayachawan P, McDonald A (2008). Fatigue and fracture properties of yttria partially stabilized zirconia crown systems. Dent Mater.

[31] Saito A, Komine F, Blatz MB, Matsumura H (2010). A comparison of bond strength of layered veneering porcelains to zirconia and metal. J Prosthet Dent.

[32] Rosentritt M, Steiger D, Behr M, Handel G, Kolbeck C (2009). Influence of substructure design and spacer settings on the in vitro performance of molar zirconia crowns. JDent.

